# “Non alcoholic fatty liver disease and eNOS dysfunction in humans”

**DOI:** 10.1186/s12876-017-0592-y

**Published:** 2017-03-07

**Authors:** Marcello Persico, Mario Masarone, Antonio Damato, Mariateresa Ambrosio, Alessandro Federico, Valerio Rosato, Tommaso Bucci, Albino Carrizzo, Carmine Vecchione

**Affiliations:** 1 Internal Medicine and Hepatology Unit, PO G. Da Procida—AOU- San Giovanni e Ruggi D’Aragona, University of Salerno, Via Salvatore Calenda 162, CAP: 84126 Salerno, Italy; 2 Vascular Physiopathology Unit IRCCS, INM Neuromed, Pozzilli, IS Italy; 3 Hepato-Gastroenterology Division, University of Campania “L. Vanvitelli”, Naples, Italy; 4 Internal Medicine and Hepatology Department, University of Campania “L. Vanvitelli”, Naples, Italy; 5 Department of Medicine and Surgery, University of Salerno, Salerno, Italy

**Keywords:** Non-alcoholic fatty liver disease, Endothelial dysfunction, Metabolic syndrome, Insulin resistance

## Abstract

**Background:**

NAFLD is associated to Insulin Resistance (IR). IR is responsible for Endothelial Dysfunction (ED) through the impairment of eNOS function. Although eNOS derangement has been demonstrated in experimental models, no studies have directly shown that eNOS dysfunction is associated with NAFLD in humans. The aim of this study is to investigate eNOS function in NAFLD patients.

**Methods:**

Fifty-four NAFLD patients were consecutively enrolled. All patients underwent clinical and laboratory evaluation and liver biopsy. Patients were divided into two groups by the presence of NAFL or NASH. We measured vascular reactivity induced by patients’ platelets on isolated mice aorta rings. Immunoblot assays for platelet-derived phosphorylated-eNOS (p-eNOS) and immunohistochemistry for hepatic p-eNOS have been performed to evaluate eNOS function in platelets and liver specimens. Flow-mediated-dilation (FMD) was also performed. Data were compared with healthy controls.

**Results:**

Twenty-one (38, 8%) patients had NAFL and 33 (61, 7%) NASH. No differences were found between groups and controls except for HOMA and insulin (*p* < 0.0001). Vascular reactivity demonstrated a reduced function induced from NAFLD platelets as compared with controls (*p* < 0.001), associated with an impaired p-eNOS in both platelets and liver (*p* < 0.001). NAFL showed a higher impairment of eNOS phosphorylation in comparison to NASH (*p* < 0.01). In contrast with what observed in vitro, the vascular response by FMD was worse in NASH as compared with NAFL.

**Conclusions:**

Our data showed, for the first time in humans, that NAFLD patients show a marked eNOS dysfunction, which may contribute to a higher CV risk. eNOS dysfunction observed in platelets and liver tissue didn’t match with FMD.

## Background

Non-Alcoholic Fatty Liver Disease (NAFLD) is represented by two clinical features: Non-Alcoholic Fatty Liver (NAFL) (namely “steatosis”), and Non-Alcoholic-Steato-Hepatitis (NASH) (namely: “steatohepatitis”). NAFLD is a unique “challenge” for the hepatologists and has increased worldwide in the last few years, due to the changes in dietary habits and increased sedentary lifestyle. Consequently NAFLD can be considered one of the most frequent liver diseases in the world [1]. It is generally considered a “benign disease” with low rates of progression to fibrosis, cirrhosis and hepatocellular carcinoma (HCC) [2]. Nevertheless, because of the high number of affected patients, the prevalence of related cirrhosis gradually increased, and actually it represents the third cause of liver transplantation in the USA. Moreover, even if the incidence of HCC in NAFLD patients is lower than that in HCV/HBV cirrhotic patients, the absolute burden of NASH-related HCC is higher, due to the higher number of patients with NAFLD [3]. It is very likely that the importance of this disease will continue to increase in the future, when the new therapies and prevention programs for hepatitis C and B will further reduce the size of viral infections of the liver. For these reasons, to recognize the mechanisms underlying its onset and progression is very important. Even if a lot of insights on this topic have been postulated in the last few years, many aspects of the pathophysiological mechanisms underlying this disease remain to be explored. The hypothesis, risen from recent papers on animal experimental models, that found a possible linkage among microvascular abnormalities in the fatty liver, the lipid accumulation into the hepatocytes, and the fibrosis [4–6], seems to be of interest. In particular, it has been postulated that in NAFLD may be present an endothelial dysfunction that could be one of the earliest factors associated to fat accumulation and liver damage [7–9]. This finding is not totally surprising if we consider that, since its discovery, NAFLD has been widely associated to cardio-metabolic syndrome and its components: hepatic and systemic insulin resistance (IR), dyslipidemia, visceral obesity, hypertension, impaired fasting glucose, [10] and increased stroke risk [11]. Although a large number of insights on the linkage between IR and liver damage are yet unknown, what we know is that IR per se is responsible for endothelial dysfunction, for example via the imbalance of the enzymatic system of Nitric Oxide production (NO) [12]. In fact, insulin was proven to induce endothelial Nitric Oxide Synthase (eNOS) activation, resulting in vasodilation and vascular protection [13]. When IR appears, it can also lead to endothelial dysfunction, through the impairment of NO production and the inhibition of insulin-induced vasorelaxation [14], and eNOS function impairment has been widely associated to it [15]. Moreover, it was demonstrated that endothelial dysfunction with impaired NO production is involved in the progression of advanced liver diseases such as cirrhosis [16, 17], and it is associated with increased vascular resistance (resulting in portal hypertension) and hepatic stellate activation in the liver (resulting in fibrosis) [18]. All together these evidence led to consider the possibility, subsequently confirmed, that this mechanism already acts in earlier stages of murine experimental models of NAFLD-related liver damage [7–9]. Nevertheless, to our knowledge, no experimental studies have confirmed these findings on human models of NAFLD.

Flow-mediated vasodilation (FMD) of the brachial artery by means of ultrasonography [19] is a well known test to assess systemic endothelial function in humans [20, 21] and its significant clinical value is based on the assumption that the vasodilatory capacity of a vessel during post-ischemic hyperemia depends on a preserved NO synthesis and release [19]. In non-cirrhotic subjects, cardiovascular risk factors impair FMD due to the oxidative stress-induced systemic endothelial dysfunction [22, 23].

The aim the of the present study is to try to demonstrate that eNOS derangement together with FMD impairment are associated with NAFLD.

## Methods

Fifty-four consecutive patients (38 males, 16 females), coming from January 2014 to April 2015 to our tertiary center of Hepatology for the evaluation of their liver disease by liver biopsy, were enrolled in the present prospective case-control study. Inclusion criteria were histological diagnosis of non alcoholic steatosis (NAFL) and/or steatohepatitis (NASH). Exclusion criteria were the presence of any other concomitant liver disease: viral infections (HCV, HBV, HIV), autoimmunity, drug hepatitis, unsafe alcohol consumption (more than 20gr/day) or neoplastic diseases. The control group consisted of healthy volunteers matched for age and sex with the study population was recruited by a local blood bank.

Patients were divided into two groups according to the liver biopsy results: 1) simple steatosis (NAFL), 2) steatohepatitis (NASH). The results obtained in the individual groups were compared between each other and with the control group. NASH patients were also stratified by fibrosis degree at liver biopsy.

- *Clinical evaluation*: Of each patient (and control) clinical history with alcohol consumption and smoking habits registration, physical examination, arterial pressure, waist circumference, body mass index (BMI), blood glucose, total and fractioned cholesterol, triglycerides, AST, ALT, GGT, ALP, complete blood count, metabolic syndrome evaluation by NCEP-ATPIII criteria were recorded [24].
- *Liver disease assessment*: An abdomen ultrasound examination with the evaluation of the liver echo pattern of liver steatosis was performed by a skilled ultrasonographist at the time of enrollment [25]. On the basis of the clinical status of the patient and the good clinical practice behaviour, liver tissue samples were collected by performing a hepatic percutaneous biopsy with Surecut 17G needles, via the intercostal route using an echo-guided method. Liver specimens were used for histological examination if they were at least 1.5-cm long and contained >5 portal spaces. Biopsies were evaluated by using both the Kleiner score [26] for necroinflammation grading and fibrosis staging and the Brunt score [27] for the presence and extent of steatosis by a skilled pathologist. Each patient, and control, was included in the study after signing a written informed consent.
- *Ethics statement*: The present study was approved by our local ethical committees (Ethical Committee of Istituto Neurologico Mediterraneo IRCCS Neuromed for experimental animals and Ethical Committee Campania Sud for patients and control subjects). The study protocol is in accordance with the ethical guidelines of the 1975 Declaration of Helsinki. All animals received humane care according to the criteria outlined in the “Guide for the Care and Use of Laboratory Animals” prepared by the National Academy of Sciences and published by the National Institutes of Health (NIH publication 86-23 revised 1985).

### Evaluation of vasorelaxation activity inhibition

*Platelet isolation and isolated vessel study:* The assessment of the eNOS function was performed by evaluating vasorelaxation activity induced on isolated mice vessels by platelet-rich plasma (PRP) obtained by peripheral blood samples of patients and controls, activated with insulin. The response to vasodilator supernatants obtained through the stimulation of platelets with insulin was examined after achieving a preconstricted tone with increasing doses of phenylephrine on isolate mice aorta rings. These were mounted between stainless steel triangles. The whole method has been already described by our group in a study on another patients’ setting [28].

### Immunoblotting

After the isolation, platelets were solubilized in lysis buffer. Then, the supernatants were used to perform immunoblot analysis with anti-phospho-eNOS S1177 (Cell Signaling, rabbit polyclonal antibody 1:800); anti-total-eNOS (Cell Signaling, mouse mAb 1:1000), anti-iNOS (BD Laboratories cod.610599 mAb 1:800), anti-pAkt (Thr 308, Santa Cruz sc-135650 mAb 1:800), anti-Akt (Santa Cruz sc-56878 mAb 1:800) and β-actin (Cell Signaling, mouse mAb 1:2000). The whole method has been previously described [28].

### Immunohistochemistry for hepatic eNOS

Sections of liver tissue were immunostained with p-eNOS serin 1177 antibody (abcam ab75639). Briefly, 3-L thick slices of formalin-fixed, paraffin-embedded liver were mounted on slides. The slides were deparaffinized, followed by suppression of endogenous peroxidase activity by immersion in PBS containing 2% H_2_O_2_ for 30 min. Nonspecific binding was blocked with 10% horse serum in PBS at room temperature for 1 h. The sections were washed in PBS with 0.05% Tween-20 thrice for 2 min each time, followed by incubation overnight at 4 °C with mouse anti-p-eNOS antibody [1:50] in PBS containing 4% horse serum. The sections were washed in PBS twice for 2 min each time, followed by incubation for 1 h at room temperature with biotinylated goat anti-mouse IgG [1:200] in PBS containing 1.5% horse serum. Then, the slides were incubated with avidin-biotin peroxidase conjugate for 30 min at room temperature; the coloured reaction product was developed by incubation for 7 min with 0.05% diaminobenzidine in 0.01% H_2_O_2_ in PBS. Negative controls were carried out under the same conditions by using mouse IgG instead of p-eNOS antibody.

### FMD of brachial artery by ultrasound

FMD measurements were performed by a single operator trained in this method, holding an intra-observer variability less than 5%. The technique was carried out following the published guidelines [29]. In particular, patients, after a fasting of at least 8 h, were examined after a 20 min rest in a quiet and darkened room. They were instructed of avoiding smoking, drinking coffee and/or alcohol, and eating high-fatty food within the previous 12 h. A standard cuff was positioned around the right arm, 2 in. below the antecubital fossa, with the patient in the supine position.

To acquire images of the right brachial artery, a 10-MHz linear probe connected to a Hi Vision Preirus ultrasound system (Hitachi Hi Vision Preirus, Hitachi Medical Corporation, Tokyo, Japan) was used. Baseline images were obtained for 2 min, then the right brachial artery was occluded by inflating the cuff to above 250 mmHg, and kept inflated for 5 min. Subsequently, the cuff was deflated and images of the right brachial artery were captured. FMD was calculated with the following formula: (maximum diameter-baseline diameter)/ baseline diameter) × 100 [30].

### Statistical analysis

Statistical analyses were performed by using the Statistical Program for Social Sciences (SPSS®) ver.16.0 for Macintosh® (SPSS Inc., Chicago, Illinois, USA). Student *t*-test and Mann-Whitney *U* test were performed to compare continuous variables, chi-square with Yates correction or Fisher-exact test to compare categorical variables. Univariate and multivariate analyses were performed to test independent variables affecting the endothelial dysfunction, by performing ANOVA, linear regressions and binary logistic regressions, where applicable. Statistical significance was defined when “*p* < 0.05” in a “two-tailed” test with a 95% Confidence Interval.

*Sample size calculation:* in order to find an adequate sample size, we performed an interim analysis on the first 18 patients (10 NASH and 8 NAFLD) and enrolled controls, that we used as “calibration set”. The sample size was calculated on the basis of the results of vasorelaxation experiment. The number needed to elicit a statistical difference between NAFL and NASH patients, with a power of 0.9 and an alpha error of 0.01 in a two sided test with 95% CI, was of 20 subjects per arm. We decided to include all the 54 cases enrolled in the present study to further improve the reliability of our results.

## Results

Of the 54 patients 21 (38, 8%) had NAFL and 33 (61, 7%) had NASH according to histological diagnosis. No statistically significant differences were found between the two groups for age, sex, BMI, ALT, prevalence of hypertension, diabetes, dyslipidemia, obesity and metabolic syndrome. The only statistical difference was found in HOMA score and insulin levels (*p* < 0.001 both), see Table 1. Six patients in NASH group at liver biopsy were found to have the histological features of cirrhosis. Due to the fact that liver cirrhosis may represent perse a cause of endothelial derangement [31], we performed the vasoreactivity and NOS evaluations with and without the samples derived from cirrhotic patients, and we did not find any significant difference in the results of the various experiments. The data here presented belong to the non-cirrhotic patients.

**Table 1:** Clinical characteristics of our study population and controls

Variable	Controls *n*: 15	Overall NAFLD *n*: 54	*p*	NAFL *n*: 21	NASH *n*: 33	*p*
Age	45,55 ± 10,24	49,89 ± 1,05	0.176	48,58 ± 10,874	50.27 ± 10.254	0.566
Sex M/F	10/5 [66,7%/32,3%]	38/16 [70,4%/27,8%]	0.784	14/7 [66,7%/33,3%]	24/9 [72,7%/27,3%]	0.634
BMI	22,91 ± 2,76	30,17 ± 3,86	<0.001	29,51 ± 3,31	30,6 ± 4,21	0.320
Hypertension	0	21 [38,9%]	0.005	7 [33,3%]	14 [42,4%]	0.521
Diabetes	0	11 [22,2%]	0.057	4 [19,0%]	7 [21,2%]	0.847
Dyslipidemia	0	35 [64,8%]	<0.001	16 [76,2%]	19 [57,6%]	0.163
Obesity	3 [20,00%]	29 [53,7%]	0.021	12 [57,1%]	17 [51,5%]	0.686
Insulin	–	17,65 ± 5,95	–	14,08 ± 2,06	21,18 ± 6,98	<0.001
HOMA	–	4,36 ± 1,72	–	3,10 ± 0,08	5,24 ± 1,58	<0.001
Metabolic Syndrome	0	19 [35,2%]	0.007	7 [33,3%]	12 [36,4%]	0.820
Cirrhosis	0	6 [11,1%]	0.177	0	6 [18,2%]	0.077
ALT	26,44 ± 9,56	67,56 ± 44,37	<0.001	66,00 ± 43,65	68,33 ± 46,64	0.085

### Histological evaluation

As reported in methods, the diagnosis of NAFL or NASH was performed by applying the Kleiner score to the collected liver samples [26]. In particular, a patient was defined to have NASH instead of simple NAFL if its “NAFLD Activity Score” was ≥ 5, see Table 2.

**Table 2:** Histological scores [mean ± SD] according to Kleiner score in NAFLD patients

	Overall	NAFL	NASH	*p*	95% CI
Steatosis [0–3]	1.8 ± 0.9	1.4 ± 0.7	2,1 ± 1.1	0.0122	−1.241/−0.159
Lobular Inflammation [0–2]	1.9 ± 1.0	0.9 ± 0.7	2.2 ± 1.2	0.0001	−1.881/−0.719
Hepatocellular Ballooning [0–2]	1.16 ± 0.7	0.7 ± 0.2	1.9 ± 1.1	0.0001	−1.688/−0.712
Fibrosis [0–4]	1.9 ± 0.9	0.5 ± 1.0	2.9 ± 0.9	0.0001	−2.926/−1.874
NAS*mean ± SD (median/range)	4.7 ± 0.9 (4.5/2–11)	3.6 ± 1.2 (3.0/2–4)	6.9 ± 1.4 (6.5/5–11)	0.0001	−2.252/−1.348

*NAFLD activity score: in parenthesis median and range

### Vasorelaxation induced by platelet-supernatant is altered in steatosis/steatohepatitis

**Fig. 1:**
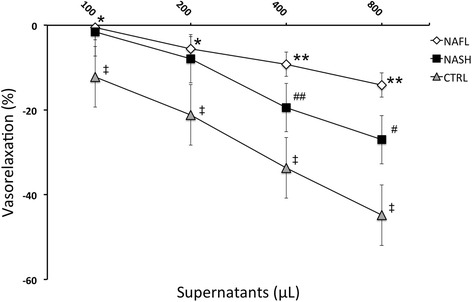
Dose-response curves of phenylephrine-precontracted aorta rings to supernatants derived from stimulated platelets isolated from NASH, Steatosis patients (“NAFL”) or Control subjects. Steatosis vs. controls:*, *p* < 0.05; **, *p* < 0.001; NASH vs. Steatosis: #, *p* < 0.05; ##, *p* < 0,001; NASH vs. controls: ‡, *p* < 0.05

The supernatant from stimulated human platelets induced a rapid dose-dependent relaxation of mice aorta rings and was abolished by NOS inhibitor, L-NAME (data not shown), clearly demonstrating the involvement of NO signalling in supernatant vascular action. Interestingly, our data demonstrate that the vasorelaxant effect, induced from platelet supernatants, was markedly reduced in NASH/NAFL patients when compared to control subjects. Moreover, the vasorelaxation induced from platelet supernatants from NAFL patients was significantly reduced if compared to that obtained from NASH subjects (Fig. 1).

### Platelet eNOS-phosphorylation is impaired in steatosis/steatohepatitis

**Fig. 2:**
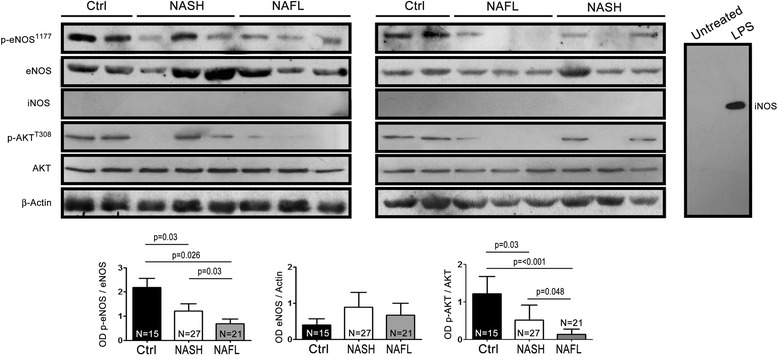
Representative immunoblotting of eNOS phosphorylation at serine residue 1177 and Akt in threonine 308 in platelets and relative densitometric analysis for p-eNOS (*left panel*), p-Akt (*central panel*) and eNOS (*right panel*)

Levels of eNOS phosphorylation (p-eNOS) decreased in platelets of NASH patients compared to platelets of control subjects. Interestingly, in platelets of NAFL subjects there is an important impairment of eNOS phosphorylation both versus control subjects and versus NASH patients. Conversely, eNOS expression did not change among all the groups. In addition, no changes in the levels of β-actin were observed between all the groups, whereas levels of Akt phosphorylation (pAkt) decreased as much as eNOS levels (Fig. 2). These results reproduce and confirm those found on vasorelaxation inhibitory mechanism (see Fig. 1).

To evaluate the involvement of iNOS in our results, we performed its expression in our platelet samples. Our data revealed an absence of iNOS expression in our experimental conditions.

In agreement, It has been demonstrated that iNOS expression is absent in platelets [32]. By contrast, iNOS expression was enhanced after LPS treatment in mice vessels (see Fig. 2), as previously demonstrated [9].

### eNOS phosphorylation is reduced in liver from steatosis/steatohepatitis

**Fig. 3:**
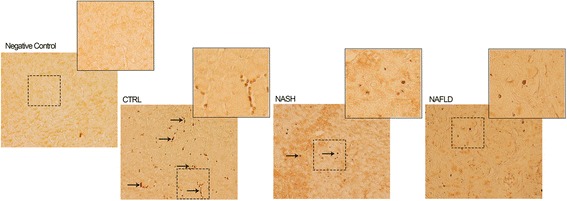
Immunohistochemistry of p-eNOS in the liver. Immunohistochemistry was performed on liver from three experimental condition: CTRL [control], NASH and Steatosis (“NAFL”). Insets represent higher magnification micrographs of p-eNOS immunoreactivity

Immunohistochemical data showed that liver samples collected from health control subjects, showed a marked staining for p-eNOS in S1177 compared to liver samples obtained from both NASH and NAFL subjects. Interestingly, the pattern of phosphorylated eNOS levels is equivalent to that found, using western blot analyses, in platelets from the same subjects (Fig. 3).

### Flow-mediated dilation (FMD) of the brachial artery by ultrasound

**Fig. 4:**
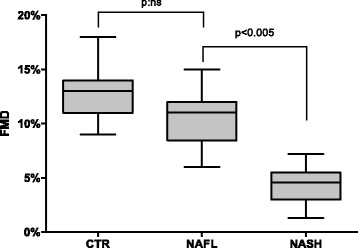
Flow-mediated dilation evaluation of patients and controls in our study population

FMD resulted lower, although not significant (*p* = 0.534), in NAFL patients if compared to controls and differed between NAFL and NASH, showing a statistically significant FMD decrease (10.72 ± 0.89 vs 4.34 ± 1.5 *P* <0.0001) in NASH patients (Fig. 4). This, apparently, seems to contradict data reproduced by platelet supernatant evoked vasorelaxation on mice aorta rings (see Fig. 1) and possible interpretation of discrepancy will be reported in discussion.

## Discussion

The present study shows, for the first time in humans, that an impaired eNOS function may be present in NAFL and NASH. This could lead to a worse NO production and an impairment of platelet-mediated vasorelaxation induction. A novel “dynamic” method, already proven to be an affordable and reliable tool to investigate vascular and platelet eNOS function [33, 34], was therefore used. Data here reported show that platelet-mediated vasorelaxation effect is repressed in NAFL and NASH patients. Since this effect is suppressed by NOS inhibitors (i.e. L-NAME), it is clear that NO signalling is involved in platelet-mediated vascular activity. This novel approach may represent a reliable tool, available to study the endothelial function in NAFLD patients. In particular, it has the benefit of being substantially constituted by a simple blood sample analysis that can be easily repeated, and might be easily used to assess the course of the disease and/or the results of eventual therapies.

The association between eNOS impairment and vascular reactivity seems to be related to the significantly reduced functional fraction of eNOS, the Serine-1177 phosphorylated-eNOS, either in platelets derived by peripheral blood samples and in liver tissue specimens of NAFL and NASH patients, if compared to healthy controls. This is in line with previous studies conducted on murine experimental models of NAFL, in which e-NOS dysfunction was also hypothesized [7, 8]. Thus, eNOS derangement, leading to NO reduction and, consequently, to endothelial dysfunction, might represent one of the main pathophysiological mechanisms involved in the liver damage by fat accumulation. In this regard, recent studies, both in humans and animal models of NASH, demonstrated derangements in microvascular functionality of the liver tissue [4, 5]. Moreover, a sinusoidal dysfunction associated with lipid accumulation in hepatocytes together with collagen deposition in the space of Disse was also highlighted [5]. Furthermore, in human liver specimens, iNOS expression, measured by immunohistochemistry, was correlated to the histological activity index and fibrosis in chronic viral hepatitis [35].

One of the main mechanisms promoting NO production through the activation of eNOS is the insulin signaling pathway [11, 12]. Insulin Resistance [IR], widely demonstrated in NAFLD, might be the main trigger of eNOS dysfunction that, therefore, might play a crucial role in the onset of NAFLD. Liver sinusoidal endothelial cells act in the same way of the other common endothelial cells. Their function is crucial to defend the tissue from inflammation and fibrosis [36, 37], therefore an impairment of their activity may promote, or worsen, the inflammatory state, known as “low-grade inflammation”, which underlies NAFLD onset and progression [38]. This seems to be supported by the fact that liver endothelial dysfunction is significantly associated to advanced liver diseases (of every etiology) and portal hypertension [39–41].

In this way, liver eNOS dysfunction might be one of the earliest “triggers” of liver damage and an important responsible for fibrosis progression.

It has been largely demonstrated in various experimental studies that the endothelial damage and derangement of endothelial regulatory mechanisms represent the pathophysiological basis of cardiovascular disease (CVD), and probably, in this regard, eNOS and iNOS are the most important elements. It is known that NAFLD may represent an independent risk factor for CVD as demonstrated in several studies, also when adjusting for the “classical” CVD risk factors, such as hypertension, dyslipidemia, obesity, and diabetes [42–45]. Moreover, a wide variety of papers demonstrated that NAFLD is associated with indirect markers of microvascular dysfunction [46–49]. Here-reported eNOS dysfunction in NAFLD patients might represent a significant contribution on the comprehension of the pathophysiological linkage between NAFLD and CVD.

Another point raised by our results which deserves a discussion is that the simple steatosis (NAFL), which represents the early stage of the disease, seems to be associated to a worse eNOS impairment compared to steatohepatitis (NASH). This was confirmed both by the dynamic evaluation on mice aorta rings, on which a more intense inhibition of the vasorelaxation was found in NAFL, and by Immunoblot assays, in which it was clearly demonstrated that NAFL patients had significantly lower levels of s1177-p-eNOS if compared to both NASH and healthy controls. Finally, the immunohistochemical evaluation of p-eNOS on the liver tissue samples confirmed this trend. On the other hand, clinical evaluation of endothelial dysfunction, measured via FMD, has shown to be worse in NASH than NAFL patients, confirming a worse endothelial dysfunction and, therefore, a higher risk of cardiovascular disease in NASH subjects which has already been largely reported [22, 23]. This apparent discrepancy between the confirmation of a higher cardiovascular risk derived from a worse endothelial reactivity “in-vivo”, and a “less-worse” eNOS function in NASH patients, is not explained by any pharmacological influence that may have altered the results. In fact, even if it is known that some drugs, such as insulin sensitizer metformin [50–52], PPAR-gamma agonists thiazoledinediones [53] calcium channel blockers [54], ACE inhibitors and ARBs [55] may have a positive effect on eNOS expression, no differences were found in the use of these drugs between NAFL and NASH patients in our population. Therefore, if we can reasonably exclude any pharmacological interference, a plausible hypothesis explaining our results might be represented by the fact that, due to the worse insulin resistance, NASH subjects have a higher circulating insulin level if compared to NAFL patients, as demonstrated by insulin levels and HOMA scores (see Table 1). This “hyper-insulinemia” could lead to a “partial recovery” of the eNOS phosphorylation which, in turn, may explain the slightly better eNOS activity of NASH patients compared to NAFL ones. Interestingly, this last hypothesis is supported by our finding showing that Akt impairment is more evident in NAFL as compared to NASH. Another possible speculation could concern the fact that in a chronic disease such as NASH, a large amount of cytokines are released. Some of them (ie. VEGF, TNF-alpha and TGF-beta) were proven to have effects on eNOS expression and activity [55]. In such a “signaling storm” a mechanism of positive feedback could be postulated, and lead to a partial recovery of eNOS phosphorylation. Moreover, it has to be pointed out that not even the histological finding of a cirrhosis influenced these results: even if we presented the data without the six NASH patients with histological diagnosis of fibrosis, we carried out also the experiments including these samples, and nothing changed in terms of statistical significance. Finally, the discrepancy between clinical and laboratory results may also be explained by the higher redox status in NASH. In fact, the presence of inflammatory status leads to ROS production that reduces both bioavailability and NO production. In particular, superoxide anion (O2-) can blind NO, producing peroxynitrite (ONOO-), an highly toxic reactive oxygen species, and can interact with tetra-hydro-bio-pterin determining the decoupling of eNOS. These speculative interpretations need to be warranted by further studies, currently performed in our lab. However, the mechanisms underlying the endothelial dysfunction, here reported, support the idea that the endothelial dysfunction might play a crucial role in the pathological “first hit”, responsible for fat accumulation in NAFLD, apparently dissociated by the following chronic inflammatory process that per se might beresponsible for the evolution, through fibrosis, to more severe steatohepatitis. Sophisticated and intriguing hypotheses of pathophysiological mechanism (s) modifying the endothelial function in NAFL and NASH patients represent the main message of the present study NASH patients have a higher cardiovascular risk, as documented by FMD measurements.

### Study limitations

The present study has some limitations. Firstly, the present data need to be validated on larger series. Then, it is already well known that vascular dysfunction involves not only eNOS, but also the inducible isoform of Nitric Oxyde Synthase (iNOS) which was not present in the human platelets evaluated, and it may be investigated in future studies using another experimental model. Finally, these data should be investigated together with the evaluation of direct markers of liver inflammation and fibrosis (such as TNF-alpha, TGF-beta and collagenase) in order to find any supposed direct correlation between endothelial dysfunction and liver disease progression.

## Conclusions

Data here reported support the idea that IR-associated eNOS dysfunction may represent a peculiar and essential mechanism of liver damage in NAFLD, that might also represent a pathological linkage between NAFLD and CVD. Moreover, supporting the pathogenic hypothesis of “multiple parallel hits”, data here reported seem to demonstrate that eNOS dysfunction might be regarded as an essential pathophysiological feature of the “first hits” of the chronic progressive process of NAFLD/NASH.

## Abbreviations

ED: Endothelial dysfunction
eNOS: Endothelial nitric oxyde synthase
HOMA: Homeostasis Model Assessment
iNOS: Inducible nitric oxyde synthase
IR: Insulin resistance
NAFL: Non alcoholic fatty liver
NAFLD: Non alcoholic fatty liver disease
NAS: NAFLD Activity score
NASH: Non alcoholic steato-hepatitis
NO: Nitric oxyde
