# Correction to: “Non alcoholic fatty liver disease and eNOS dysfunction in humans”

**DOI:** 10.1186/s12876-017-0671-0

**Published:** 2017-11-08

**Authors:** Marcello Persico, Mario Masarone, Antonio Damato, Mariateresa Ambrosio, Alessandro Federico, Valerio Rosato, Tommaso Bucci, Albino Carrizzo, Carmine Vecchione

**Affiliations:** 1 Internal Medicine and Hepatology Unit, PO G. Da Procida—AOU- San Giovanni e Ruggi D’Aragona, University of Salerno, Via Salvatore Calenda 162, CAP: 84126 Salerno, Italy; 2 Vascular Physiopathology Unit IRCCS, INM Neuromed, Pozzilli, IS Italy; 3 Hepato-Gastroenterology Division, University of Campania “L. Vanvitelli”, Naples, Italy; 4 Internal Medicine and Hepatology Department, University of Campania “L. Vanvitelli”, Naples, Italy; 5 Department of Medicine and Surgery, University of Salerno, Salerno, Italy

## Correction

After publication of the original article [1], it was noticed that one of the affiliations of author Carmine Vecchione was missing.

The updated and correct author affiliations of the original article [1] are presented in this erratum.
